# Comparison of nicotine dependence between single and multiple tobacco product users among South Korean adults

**DOI:** 10.18332/tid/145899

**Published:** 2022-02-25

**Authors:** Youn Huh, Cheol Min Lee, Hong-Jun Cho

**Affiliations:** 1 Department of Family Medicine, Uijeongbu Eulji Medical Center, Eulji University School of Medicine, Uijeongbu, Republic of Korea; 2 Department of Family Medicine, Seoul National University Hospital Healthcare System Gangnam Center, Seoul, Republic of Korea; 3 Department of Family Medicine, Asan Medical Center, University of Ulsan College of Medicine, Seoul, Republic of Korea

**Keywords:** electronic cigarette, heated tobacco products, multiple tobacco user, nicotine, nicotine dependence

## Abstract

**INTRODUCTION:**

The relationship between nicotine dependence and the use of multiple tobacco products, such as heated tobacco products (HTPs), electronic cigarettes (ECs), and combustible cigarettes (CCs), is not well investigated. We evaluated nicotine dependence symptoms among South Korean adults among single and multiple tobacco product users.

**METHODS:**

We conducted an online survey involving 7000 adults aged 20–69 years in November 2018 and compared the nicotine dependence among single, dual, and triple use of tobacco products. Nicotine dependence was measured for ‘time to the first use of tobacco products within 5 min’, ‘awaking at night’, ‘strong craving’, ‘uncontrollable urge to smoke’, and ‘irritability or restlessness’. Adjusted odds ratios (AORs) and 95% confidence intervals (CIs) for nicotine dependence symptoms based on the number of tobacco products were estimated using multivariable logistic regression analysis.

**RESULTS:**

The current prevalence of use of tobacco products was 27.5%. Proportion of dual and triple uses were: 28.3%, 13.7% for CCs; 36.5%, 50.2% for ECs; and 54.0%, 33.1% for HTPs. Nicotine dependence tended to be higher as the number of tobacco products used increased for most measures, except for ECs with the measure ‘time to first use of tobacco products within 5 min’. The ORs of ‘awaking at night’ increased approximately three times for dual users (OR=2.87; 95% CI: 1.29–6.39, for current EC users) and seven times for triple users (OR=7.24; 95% CI: 3.66–14.31, for current HTP users) compared to that for single users.

**CONCLUSIONS:**

Multiple tobacco product users reported higher nicotine dependence symptoms than single users. High nicotine dependence of multiple tobacco product users may hamper the future cessation of tobacco products, which can be challenging for future tobacco control policies in South Korea.

## INTRODUCTION

In 2021, the World Health Organization reported that the worldwide prevalence of smoking had decreased from 22.7% in 2007 to 17.5% in 2019^1^. In Korea, the adult smoking prevalence decreased from 35.2% in 1998 to 20.2% in 2019^2^. The number of Korean adults who use electronic cigarettes (ECs) has consistently increased from 2.3% in 2016 to 3.3% in 2019 since e-cigarette (EC) introduction to the Korean market in 2007^2^. In addition, heated tobacco products (HTPs) are fast expanding their market share in South Korea. HTPs have successfully assimilated in the market and increased interest among smokers since IQOS by Philip Morris International, a leading HTP brand, was launched in South Korea in July 2017. HTPs accounted for 2.2% of total tobacco sales in the first year of their launch, and the prevalence of HTP use in the last month among adults was 5.1% in 2019^2,3^. Furthermore, the advent of new tobacco products has altered tobacco consumption patterns; 27.4% of adult Korean tobacco product users were current dual users of combustible cigarettes (CCs), HTPs, and ECs, and 12.4% were current triple users^4^.

Nicotine is a highly addictive substance that can interfere with brain development. Nicotine dependence has been described as a combination of neurobiological symptoms and learned behaviors associated with repeated nicotine self-administration^5^. Dependence characteristics are loss of cognitive control, including strong cravings for tobacco, and using tobacco to cope with difficult situations^6^. Tobacco products vary in nicotine content, route of administration, constituent ingredients, and behavioral patterns of persistent use; therefore, cigarette-focused instruments may not effectively reflect nicotine dependence in multiple tobacco users^7,8^. There are many nicotine dependence measurements for cigarettes. Several measures including the Fagerström Test for Nicotine Dependence (FTND)^9^, Wisconsin Inventory of Smoking Dependence Motives (WISDM)^10^, and Nicotine Dependence Syndrome Scale (NDSS)^11^ can elucidate the relationship between CCs and nicotine dependence symptoms. Moreover, there was also an attempt to apply nicotine dependence measurements for CCs to smokeless cigarettes^7^. In a study comparing the above nicotine dependence measurements, 16 out of 24 items of nicotine dependence to include loss of control, craving, tolerance, and automaticity, can be used in a known instrument to assess nicotine dependence across different kinds of tobacco products users^12^. It suggests that some nicotine dependence measurements also can be used to measure nicotine dependence on tobacco products other than CCs.

Among tobacco product users in the US adult population, multiple tobacco product users had higher nicotine dependence symptoms than single users for EC, cigar, pipe, and hookah^12^. In that study, single-EC users had the lowest nicotine dependence symptoms, while single-CC users and dual users of CCs and ECs had similarly high nicotine dependence symptoms^12^. However, when comparing nicotine dependence between single-CC users and dual users of CC and EC, the nicotine dependence symptoms were more elevated in single-CC users than in dual users of CCs and ECs^13^. Most of the studies on nicotine dependence were conducted among adults in the US^14^ and included tobacco products such as CCs, ECs, smokeless tobacco, hookah, and cigars. However, studies including HTPs are limited. A recent Japanese study assessed nicotine dependence between CC users and HTP users using ‘time to first tobacco products use’ as a marker of nicotine dependence^15^. The study showed that dual users of daily CC and HTP had similar nicotine dependence as single-CC users and higher nicotine dependence than single-HTP users.

The National Adult Tobacco Survey (NATS) employs six questions to measure nicotine dependence for users of multiple tobacco products among US adults, including waking up at night, severe cravings, an uncontrollable urge to smoke, and irritability or restlessness^16^. The nicotine dependence questionnaire of NATS has been used in some studies to demonstrate nicotine dependence on multiple tobacco products^17-19^. Furthermore, the time to the first use of tobacco products may be a valid single-item measure of nicotine dependence^20^, and may be utilized for multiple tobacco product users other than CCs^19^.

Considering the increasing number of multiple tobacco products uses, we aimed to compare nicotine dependence symptoms between single and multiple tobacco product users to include CCs, ECs, and HTPs among Korean adults. Our results will help to inform tobacco control policies and encourage clinicians to offer cessation advice.

## METHODS

### Data source and study population

We conducted an online survey using a panel managed by the research company EMBRAIN (http://www.embrain.com/eng/) (Seoul, Korea), comprising 1.3 million members as of December 2018. First, we randomly selected 70000 individuals aged 20–69 years using their matching age, distributions provincially, and the 2018 national population statistics. However, from the selection of members, only 10489 responded. We excluded 2285 who exceeded our quota, 21 who did not meet the age criteria, 400 who decided not to participate in the survey, and 783 who responded incorrectly. Finally, we included 7000 individuals (2300 men and 4700 women aged 20–69 years). A questionnaire survey was conducted on 4700 women and 2300 men, with women being oversampled twofold because the prevalence of female smokers in Korea is much lower than that of male smokers. Details of the method were described elsewhere^4^.

### Assessment of the use of tobacco type

Participants were considered current CC users if they answered, ‘every day’ or ‘some days’ to the question: ‘Do you currently smoke cigarettes every day, some days, or not at all?’ with ‘more than 100 cigarettes in their lifetime’ to the question ‘What is the total number of CCs you have smoked during your lifetime?’. Those who answered ‘not at all’ and ‘more than 100 cigarettes in their lifetime’ were considered former CC users.

Participants were considered current EC users if they answered ‘Yes’ to both questions: ‘Have you ever used an EC in your life?’ and ‘Have you used an EC in the last 30 days?’. Participants who had previously used an EC but not in the last 30 days were considered former EC users.

HTP use was assessed by asking the following question: ‘Have you ever used a HTP in your life?’. Those who answered ‘yes’ to this question were additionally asked the following question: ‘Do you currently use HTP every day, some days, or not at all?’. Participants were considered current HTP users if they answered ‘every day’ or ‘some days’ to the question, whereas those who responded ‘not at all’ were considered former HTP users. HTP images and brand names (IQOS, Glo, and Lil) were provided to minimize confusion when distinguishing between HTPs and ECs.

### Assessment of nicotine dependence symptoms

We used five questions about nicotine dependence that consisted of four (awaking at night, strong craving, urge to smoke, and irritability) measures of addictions from the NATS, and time to first use of tobacco products within 5 min in the morning or waking up^17^. Symptoms of nicotine dependence were assessed by asking participants, who were currently using tobacco products, the following five questions:

1. Time to first use tobacco products: How soon after you wake up do you smoke your first CC (or EC or HTP) of the day? (≤5 , 6–30, 31–60, and >60 min).
2. Awaking at night: Do you sometimes wake up at night to have a cigarette or other tobacco product (EC or HTPs)? (Yes/No).
3. Strong craving: During the past 30 days, have you had a strong craving to use tobacco products of any kind? (Yes/No).
4. Uncontrollable urge to smoke: During the past 30 days, was there a time when you wanted to use any tobacco products (CC, EC, or HTP) so much that you found it challenging to think of anything else? (Yes/No).
5. Irritability or restlessness: During the past 30 days, how true is the following statement for you? ‘After not using tobacco for a while, I feel restless and irritable’ (Not at all true, sometimes true/often true, and always true).

### Covariates

We divided the ages into three groups: 20–34, 35–49, and 50–69 years. Monthly personal income levels (KRW) were divided into four groups: <3000000; 3000000–4999999; 5000000–6999999; and ≥7000000 (10000 Korean Won about US$8.4). Educational level was categorized into two groups depending on whether participants had received more than 16 years of education (4-year college graduate). Marital status was categorized into three groups: ‘never married or separated,’ ‘divorced or widowed’, and ‘married or cohabiting’.

### Statistical analysis

To conduct statistical analysis, we used SPSS version 24.0 (IBM Corp., Armonk, NY, USA). Because women outnumber men in the study population, the unweighted numbers and weighted percentages reflect the baseline characteristics of the participants. Continuous variables were compared using analysis of variance and categorical variables using the chisquared test. All results were presented as unweighted numbers and weighted percentages. Multivariable logistic regression analysis was performed to evaluate the association between the number of tobacco products used in each category of tobacco product type and nicotine dependence symptoms. It was adjusted for age, sex, education level, income, and marital status. Differences were considered statistically significant at p<0.05.

## RESULTS

Table 1 presents the sociodemographic characteristics of the participants. Of the 7000 participants, the mean age was 42.26 ± 11.98 years, and 61.0% had a college degree or higher. In addition, 61.4% of the total population were or had been married or cohabited. Regarding the tobacco use patterns, 14.4% were single-CC users, 0.9% were single-EC users, 1.3% were single-HTP users, 2.0% were dual users of CC and EC, 5.0% were dual users of CC and HTP, 0.5% were dual users of EC and HTP, and 3.4% were triple users of CC, EC, and HTP.

**Table 1:** Sociodemographic characteristics of the study participants, online panel, South Korea 2018 (N=7000)

*Characteristics*	*Categories*	*Men (n=2300) n (%)*	*Women (n=4700) n (%)*	*Total (n=7000) n (%)*
**Age** (years), mean ± SD		42.50 ± 12.31	42.14 ± 11.82	42.26 ± 11.98
**Age** (years)	20–34	690 (30.0)	1437 (30.6)	2127 (30.3)
	35–49	833 (36.2)	1612 (34.3)	2445 (35.3)
	50–69	777 (33.8)	1651 (35.1)	2428 (34.4)
**Income** (KRW)	<3000000	618 (26.9)	1156 (24.6)	1774 (25.7)
	3000000–4999999	817 (35.5)	1614 (34.3)	2431 (34.9)
	5000000–6999999	476 (20.7)	1078 (22.9)	1554 (21.8)
	≥7000000	389 (16.9)	852 (18.1)	1241 (17.5)
**Education level** (years)	<4 years college	752 (32.7)	2134 (45.4)	2886 (39.0)
	≥4 years college	1548 (67.3)	2566 (54.6)	4114 (61.0)
**Marital status**	Never married, separated	823 (35.8)	1567 (33.3)	2390 (34.6)
	Divorced, widowed	78 (3.4)	223 (4.7)	301 (4.1)
	Married, cohabiting	1339 (60.8)	2910 (61.9)	4309 (61.4)
**Tobacco use status**	Never users of any tobacco products	760 (33.0)	3906 (83.1)	4666 (58.1)
	Former users of any tobacco products	527 (22.9)	277 (5.9)	804 (14.4)
	Single-CC users	541 (23.5)	248 (5.3)	789 (14.4)
	Single-EC users	27 (1.2)	29 (0.6)	56 (0.9)
	Single-HTP users	44 (1.9)	33 (0.7)	77 (1.3)
	Dual users of CC and EC	72 (3.1)	39 (0.8)	111 (2.0)
	Dual users of CC and HTP	196 (8.5)	74 (1.6)	270 (5.0)
	Dual users of HTP and EC	13 (0.6)	20 (0.4)	33 (0.5)
	Triple users of CC, HTP and EC	120 (5.2)	74 (1.6)	194 (3.4)

CC: combustible cigarette. EC: electronic cigarette. HTP: heated tobacco product. KRW: 10000 Korean Won about US$8.4.

Table 2 presents the proportion of the type of tobacco product use among any type of tobacco product users. Of 1364 current CC smokers, the proportion of single, dual, and triple users was 58.0%, 28.3%, and 13.7%, respectively. Of 394 current EC users, the proportion of single, dual, and triple users was 13.3%, 36.5%, and 50.2%, respectively. Moreover, of 574 current HTP users, the proportion of single, dual, and triple users was 12.8%, 54.0%, and 33.1%, respectively.

**Table 2:** Proportion of types of tobacco products used among any type of tobacco product users online panel, South Korea 2018

*Type of product users*	*Number of products*	*Men n (%)*	*Women n (%)*	*Total n (%)*
**Current CC**	Single	541 (58.2)	248 (57.1)	789 (58.0)
	Dual	268 (28.9)	113 (25.9)	381 (28.3)
	Triple	120 (12.9)	74 (17.0)	194 (13.7)
**Current EC**	Single	27 (11.6)	29 (18.2)	56 (13.3)
	Dual	85 (36.5)	59 (36.4)	144 (36.5)
	Triple	120 (51.8)	74 (45.5)	194 (50.2)
**Current HTP**	Single	44 (11.8)	33 (16.7)	77 (12.8)
	Dual	209 (56.0)	94 (46.7)	303 (54.0)
	Triple	120 (32.2)	74 (36.7)	194 (33.1)

Data are presented as unweighted numbers (weighted percentages). CC: combustible cigarette. EC: electronic cigarette. HTP: heated tobacco product.

Table 3 shows the proportion of the nicotine dependence symptoms according to the type of tobacco product used. The percentage for ‘awaking at night’ symptoms among current CC smokers was 13.6% for single users, 21.1% for dual users, and 49.2% for triple users; current EC and HTP users showed similar patterns. The symptom of ‘strong craving’ was experienced by more than 50% of all user types among all categories of current smokers and increased in the order of single users, dual users, and triple users in all-type users. ‘Uncontrollable urge to smoke’ and ‘Irritable or restless’ also increased in the order of single users < dual users < triple users in all tobacco products type of users. There was no significant difference in the increase in the prevalence of ‘time to the first use of tobacco products within 5 min’ for EC users as the number of tobacco products used increased.

**Table 3:** Proportion of nicotine dependence symptoms according to types of tobacco products used and multivariable analysis of factors associated with it, South Korea 2018

*Number of products*	*Current CC users (n=1364)*		*Current EC users (n=394)*		*Current HTP users (n=574)*	
	*%*	*AOR (95% CI)*	*%*	*AOR (95% CI)*	*%*	*AOR (95% CI)*
**How soon after you wake up do you smoke your first CC (or EC or HTP) of the day?** (≤5 minutes)						
Single (Ref.)	15.3	1	25.4	1	10.9	1
Dual	15.4	1.16 (0.85–1.58)	17.3	0.65 (0.32–1.34)	16.5	1.75 (0.84–3.61)
Triple	18.9	**1.56 (1.06–2.29)**	18.9	0.63 (0.32–1.26)	18.9	2.06 (0.97–4.37)
**Do you sometimes wake up at night in order to have a cigarette or other tobacco product (EC or HTP)?** (Yes)						
Single (Ref.)	13.6	1	14.3	1	12.1	1
Dual	21.1	**1.92 (1.43–2.56)**	32.8	**2.87 (1.29–6.39)**	23.7	**2.19 (1.12–4.27)**
Triple	49.2	**7.22 (5.20–10.04)**	49.2	**5.94 (2.73–12.91)**	49.2	**7.24 (3.66–14.31)**
**During the past 30 days, have you had a strong craving to use tobacco products of any kind?** (Yes)						
Single (Ref.)	57.1	1	50.8	1	50.5	1
Dual	62.6	**1.39 (1.11–1.75)**	64.2	**1.93 (1.04–3.59)**	62.4	**1.66 (1.04–2.65)**
Triple	76.1	**2.58 (1.85–3.60)**	76.1	**2.81 (1.54–5.14)**	76.1	**3.21 (1.91–5.42)**
**During the past 30 days, was there a time when you wanted to use any tobacco products (CC, EC or HTP) so much that you found it difficult to think of anything else?** (Yes)						
Single (Ref.)	24.9	1	34.9	1	21.7	1
Dual	37.2	**1.92 (1.51–2.44)**	47.4	1.65 (0.88–3.08)	36.6	**2.11 (1.22–3.64)**
Triple	63.4	**5.39 (3.96–7.34)**	63.4	**2.90 (1.59–5.29)**	63.4	**6.39 (3.61–11.32)**
**During the past 30 days, how true is this statement for you that after not using tobacco for a while, I feel restless and irritable?** (Often and always)						
Single (Ref.)	7.6	1	3.2	1	4.3	1
Dual	7.3	1.00 (0.65–1.52)	5.2	3.71 (0.58–23.87)	8.8	2.57 (0.85–7.82)
Triple	13.0	**1.91 (1.21–3.03)**	13.0	**10.50 (1.80–61.43)**	13.0	**3.82 (1.24–11.74)**

Data are presented as weighted percentage. AOR: adjusted odds ratio; calculated by multivariable logistic regression analyses, adjusted for age, sex, education level, income, and marital status. CC: combustible cigarette. EC: electronic cigarette. HTP: heated tobacco product.

Results of the multivariable logistic regression analysis are presented in Table 3. The OR of ‘time to the first use of tobacco products within 5 min’ was 1.56 (95% CI: 1.06–2.29) among triple-CC users compared to single-CC users. For EC users, ‘time to first tobacco products use within 5 min’ was not significantly different between single and poly (dual and triple) users. The OR of ‘awaking at night’ increased two to three times for dual users (OR=1.92; 95% CI: 1.43–2.56) for current CC users, 2.87 (95% CI: 1.29–6.39) for current EC users, and 2.19 (95% CI: 1.12–4.27, for current HTP users) and increased six to seven times for triple users (OR=7.22; 95% CI: 5.20–10.04) for current CC users, 5.94 (95% CI: 2.73–2.91) for current EC users, and 7.24 (95% CI: 3.66–14.31) for current HTP users compared to that for single users. The OR of ‘strong craving’ among current tobacco users was significantly higher for dual users (OR=1.39; 95% CI: 1.11–1.75) for current CC users, 1.93 (95% CI: 1.04–3.59) for current EC users, and 1.66 (95% CI: 1.04–2.65) for current HTP users, than single users. The OR of ‘strong craving’ increased three times for triple users (OR=2.58; 95% CI: 1.85–3.60) for current CC users, 2.81 (95% CI: 1.54–5.14) for current EC users, and 3.21 (95% CI: 1.91–5.42) for current HTP users, compared to single users. The OR of ‘uncontrollable urge to smoke’ increased by two times for dual users [(OR=1.92; 95% CI: 1.51–2.44) for current CC users, and 2.11 (95% CI: 1.22–3.64) for current HTP users] and five to six times for triple users [(OR=5.39; 95% CI: 3.96–7.34) for current CC users, and 6.39 (95% CI: 3.61–11.32) for current HTP users] compared to single users, except for current EC users whose OR increased to 2.90 (95% CI: 1.59–5.29) for triple users. The OR of ‘irritable or restless’ increased only for triple users, ranging from 1.91 (95% CI: 1.21–3.03) among current CC users to 10.50 (95% CI: 1.80–61.43) among current EC users when compared to single users.

## DISCUSSION

The symptoms of nicotine dependence were more prevalent among dual and triple users than in single users, except for single-EC users who use the product within five minutes of waking up. Furthermore, the dependent symptoms were more prevalent among triple users than dual users. However, this trend was not observed in the measure ‘time to first use of tobacco products within 5 min’, particularly among current EC users.

According to other studies, users of multiple tobacco products (CC, HTP, and ECs) are significantly more nicotine dependent than single tobacco product users^6,21^. In a study of Korean adults, dual users of CCs and ECs had greater urine cotinine levels than single users of CCs^5^. In a Japanese study that included both CCs and HTPs, dual users had higher nicotine dependence than users of only HTPs when measuring nicotine dependence with the measure ‘time to first use of tobacco products within 5 min’^15^. In addition, a prior study using various measurements of nicotine dependence showed that smokers with high nicotine dependence were less likely to quit smoking CCs^22^. Therefore, the high nicotine dependence of multiple tobacco product users may hamper future cessation of tobacco products.

Several factors may explain why people who use multiple tobacco products have higher nicotine dependence than those who use a single tobacco product. First, as CC users may turn to the usage of ECs and HTPs to quit smoking, CC smokers with higher levels of nicotine dependence may further increase their nicotine dependence with the additional use of ECs and HTPs. Higher nicotine dependence, for example, is linked to a decreased likelihood of switching between tobacco products and an increase in the prevalence of multiple tobacco product users^23^. Second, if CC smokers additionally use EC or HTP, they may increase their nicotine dependence. One study showed that young adults who tried to quit smoking CCs by using ECs instead increased their nicotine dependence^24^, because some ECs can deliver nicotine at a higher speed than the nicotine dose delivered by CCs^25,26^. In a cross-sectional study among Korean adults, poly users of CC, EC, and HTPs showed a low probability of being former CC users than single-CC smokers. It suggests that poly users may quit CC smoking than CC-only users; however, a longitudinal study is required to confirm this.

We found that the nicotine dependence measurement used in our study can be applied to users of HTP and EC in addition to CC smokers. In a Japanese study, the ‘time to first tobacco product use’ was used to measure nicotine dependence of HTPs^15^. One study^19^ presented that poly users had higher nicotine dependence measured by ‘time to first tobacco product use’ than single users and showed different results from ours. In that study, the cut-off of ‘time to first tobacco product use’ was 30 minutes, unlike our study. However, in our study, contrary to other nicotine dependence measures, this measure did not show a difference between single-EC and poly-EC users, which suggests that ‘time to the first use of tobacco products within 5 min’ is not suitable for the measurement of nicotine dependence in EC users.

Findings from our study provide a basis for policy implications. Higher dependence was found among poly tobacco users and more prevalent among users of HTPs and ECs than CCs^27^. Moreover, the cessation of tobacco products for poly tobacco users is not well studied, and current smoking cessation guidelines do not deal with poly tobacco users.

### Strengths and limitations

Our study has several limitations. First, there was no representation of Korean adults using a panel of online research companies. However, the number of people of all gender, ages, and local groups surveyed corresponded to the proportion of the total population. Second, our study was based on a self-reported online survey and may be subjected to recall or measurement bias. Third, because our study used cross-sectional data, it cannot explain a causal relationship between the number of tobacco products and symptoms of nicotine addiction in adults. Fourth, respondents may not distinguish between EC and HTP because the name of EC (liquid type electronic cigarette) is like HTP (cigarette type electronic cigarette) in Korean^28^. We asked questions concerning ECs first and provided pictures and names of HTP products examples. In addition, we did not evaluate nicotine dependence with cotinine level because the survey did not include the information. Despite these limitations, our study found that the prevalence of dual- and triple-product users among current EC and HTP users was high, and nicotine dependence symptoms were higher in multiple tobacco product type users than in single tobacco product users.

## CONCLUSIONS

Our study showed that multiple tobacco product users, including CCs, ECs, and HTPs, have significantly higher nicotine dependence symptoms than single tobacco product users, except for current EC users who use the product within the first 5 minutes of their day. However, further research is needed regarding the effects of these nicotine dependence symptom measurements on smoking cessation and persistence. The study needed to subdivide dual tobacco product users such as dual users of CC and EC and dual users of CC and HTP. In addition, the high nicotine dependence of multiple tobacco product users and their stealth use of these products may hamper future cessation of the production and use of tobacco products. Therefore, tobacco control policies in South Korea should consider this to alleviate and overcome the challenges brought by tobacco use.

## Data Availability

The data supporting this research are available from the authors on reasonable request.
